# A retrospective study on comparison of clinical characteristics and outcomes of diabetic ketoacidosis patients with and without acute pancreatitis

**DOI:** 10.1038/s41598-023-31465-3

**Published:** 2023-03-16

**Authors:** Adeel Ahmad Khan, Fateen Ata, Zohaib Yousaf, Mohamad Safwan Aljafar, Mohammed Najdat Seijari, Ahmad Matarneh, Bassel Dakkak, Malik Halabiya, Bassam Muthanna, Abdul Majeed Maliyakkal, Anand Kartha

**Affiliations:** 1 Department of Endocrinology, Hamad Medical Corporation, Doha, Qatar; 2 Department of Medicine, Tower Health, West Reading, PA USA; 3 Department of Internal Medicine, Hamad Medical Corporation, Doha, Qatar; 4 Department of Gastroenterology, Hamad Medical Corporation, Doha, Qatar; 5 Department of Geriatric Medicine, University of Illinois, Chicago, USA; 6 Head of Hospital Medicine, Hamad Medical Corporation, Doha, Qatar

**Keywords:** Diseases, Endocrinology

## Abstract

The co-existence of diabetic ketoacidosis (DKA) with acute pancreatitis (AP) is associated with unfavorable clinical outcomes. However, diagnosing AP in DKA patients is challenging and often missed due to overlapping symptoms. The aim of this retrospective observational study was to compare the clinical characteristics and outcomes of patients with concomitant DKA and AP or DKA alone. Data of patients with DKA admitted between January 2015 to August 2021 to four hospitals in Qatar was extracted from the electronic health record (Cerner). American Diabetes Association criteria and Atlanta criteria were used for DKA and AP diagnosis, respectively. Independent T-test or Mann–Whitney U test was used to analyze continuous variables, whereas categorical variables were analyzed via Chi-square or Fischer exact tests as appropriate. Univariate and multivariate logistic regression models were generated to assess the correlations. A p-value of < 0.05 was considered statistically significant. Of 936 patients with DKA, 84 (9.0%) had coexisting AP. AP was most common in the Asian race (66%, p < 0.001). Patients with DKA and AP were older, had higher admission anion-gap, white cell count, hemoglobin (hb), neutrophil/lymphocyte ratio, urea, creatinine, maximum blood glucose during the episode, total cholesterol and triglyceride level (TGL) (p < 0.05). They had a lower admission venous pH and bicarbonate at 6 h. Patients in the DKA with AP group also had a longer length of stay (LOS), DKA duration and a higher rate of ICU admission (p-values ≤ 0.001). In-hospital mortality, 3-month all-cause readmission, 6-month and 12-month DKA recurrence did not differ between the two groups. Univariate logistic regression analysis showed age, Asian ethnicity, male gender, T2D, admission WBC count, hb, urea, creatinine, potassium, venous pH, bicarbonate, anion gap, total cholesterol, TGL and LDL level were significantly associated with the development of DKA with AP (p < 0.05). In multivariate logistic regression analysis, age and total cholesterol level were associated with concomitant DKA and AP (p < 0.05). Patients with concomitant DKA and AP have more severe derangement in markers of DKA severity, inflammation, kidney injury and metabolic profile, along with a longer DKA duration, LOS and requirement for ICU support compared to DKA patients without AP. This highlights the clinical significance of diagnosing the co-existence of DKA with AP, as the combination results in significantly worse clinical outcomes and greater healthcare utilization than in patients with only DKA.

## Introduction

Diabetic ketoacidosis (DKA) is a serious metabolic complication of diabetes mellitus (DM) characterized by the development of ketosis, hyperglycemia and a high anion gap metabolic acidosis^1,2^. It is more common in type 1 diabetes (T1D) patients than in type 2 diabetes (T2D). The incidence of DKA in T1D patients is reported to be 178.6/10,000 patient-years, while in T2D, it is 20/10,000 patient-years^3^. The disease is associated with a very high risk of short-term mortality, with a study reporting a mortality rate of 5.2% over a 4-year follow-up after DKA admission^4^. Acute pancreatitis (AP) is another life-threatening condition with an estimated incidence of 33.74 cases per 100,000 patient years and 1.6 deaths per 100,000 patient-years^5^. The development of concomitant DKA and AP has been reported in the literature. Recognition of the co-existence of these two entities is associated with unfavorable clinical outcomes but is challenging to recognize because of the overlapping symptoms. Abdominal pain, the most common symptom of AP, is present in up to 46% of cases of DKA and is strongly associated with more severe metabolic acidosis^6^. Interestingly, some case reports reported concomitant AP and DKA as even the initial manifestation of both T1D and T2D^7,8^.

Several studies have compared patients with coexisting DKA with AP to patients with AP alone. Yuan et al. reported a higher rate of acute kidney injury (AKI), length of stay (LOS), and severity of AP in patients of the DKA and AP group as compared to the AP group^9^. Wang et al. also reported a higher incidence of AKI, an increase in intensive care unit (ICU) admission and a higher APACHE II score in DKA with AP patients compared to AP only patients^10^. Similar results were described by Fu et al., who found statistically significant differences in ICU admission and LOS between the two groups^11^. However, data comparing patients with concomitant DKA and AP to patients with DKA alone is limited. Ma et al. compared the characteristics of DKA patients with and without pancreatitis. Their study showed a longer LOS and a higher ICU admission rate in patients with a combination of DKA and AP compared to DKA alone. However, the study only included 25 patients with coexisting AP and excluded patients with T1D^12^. Our study aimed to assess the differences in clinical characteristics and outcomes of patients with these two coexisting conditions compared to those with DKA alone. Early identification of this combination of illnesses can alert physicians to patients at high risk of developing worse clinical outcomes.

## Materials and methods

This is a retrospective, cross-sectional study and included consecutive patients with DKA diagnosis presenting to the emergency department (ED) of four hospitals of Hamad Medical Corporation (HMC), Doha, Qatar, between January 2015 and August 2021. HMC is Qatar's main tertiary healthcare provider and one of the leading healthcare systems in the Middle East.

### Inclusion criteria

All patients fulfilling the criteria of DKA were included in the study. We used American Diabetes Association (ADA) criteria for establishing the diagnosis of DKA, i.e., blood glucose greater than 250 mg/dL, high anion gap metabolic acidosis (pH < 7.3, bicarbonate < 18 mmol/L, anion gap > 10 mmol/L) and ketonemia/ketonuria^13^. Patients fulfilling the DKA diagnostic criteria were further assessed for the presence of concomitant pancreatitis using the Atlanta criteria, which includes the presence of two of the following three features: (1) Persistent upper abdominal pain, (2) Serum amylase or lipase level at least three times the upper limit of normal (3) Imaging evidence of AP^14^.

### Exclusion criteria

Patients aged less than 14 years, pregnant patients and patients with starvation or alcoholic ketosis were excluded from the analysis.

**Figure 1:**
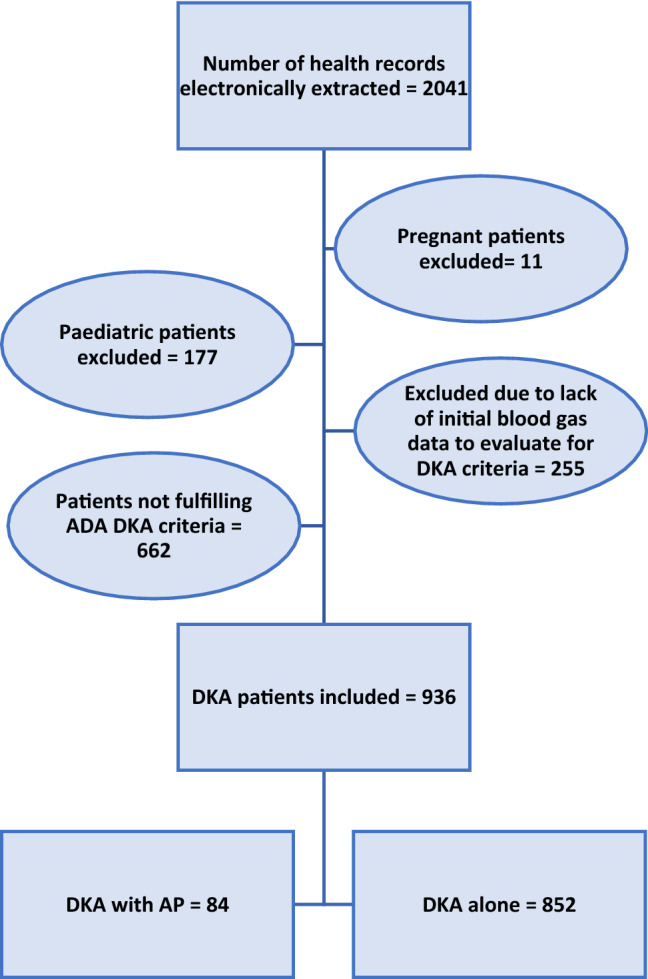
Flowchart of the process of inclusion of patients in the study.

A total of 936 patients fulfilled the criteria of DKA and were included in the study (Fig. 1).

Data were manually extracted from electronic medical records (EMR) by the research study's members. Demographic data included age, gender, ethnicity, weight, height and co-morbid conditions. All laboratory results at admission and sequential DKA related laboratory results during hospital stay (venous pH, bicarbonate, anion gap, lactate, electrolytes) were also recorded. Data related to outcome included length of stay (LOS), duration of DKA, a requirement for ICU admission, in-hospital mortality, 30-day all-cause readmission, 6-month DKA recurrence and 12-month DKA recurrence.

To ensure adequate data quality, it was collected manually from EMR and subsequently reviewed again for quality by the primary investigator. There was no missing data in laboratory variables used to establish DKA diagnosis and in variables to establish clinical outcomes of DKA. No formal method to deal with the missing data was used for other variables that had missing data.

### Statistical analysis

Continuous variables were described as either means (± standard deviation) or median (interquartile range), and an independent t-test or Mann–Whitney U test was used for comparison as appropriate. Categorical variables were described as percentages, and comparisons were performed using the chi-square and Fisher's tests. Factors associated with the development of coexisting DKA and AP were assessed using univariate and multivariate logistic regression analysis and reported as odds ratio (OR) and 95% confidence intervals (CI). A p-value of < 0.05 was considered significant. Stata version 17 was used for analysis.

### Ethical declaration

The study is original and is not under consideration for publication in another journal. The study has been approved by the Medical Research Centre (MRC) at Hamad Medical Corporation, Qatar. All methods were performed in accordance with the relevant guidelines and regulations and according to the principles laid down in the Declaration of Helsinki. All the authors reviewed and approved the final manuscript.

### Informed consent

Due to the nature of this retrospective study and the preserved anonymity of patients, a waiver of informed consent was obtained from the Medical Research Centre (MRC) at Hamad Medical Corporation, Qatar.

## Results

### Baseline characteristics of the study population

A total of 936 patients fulfilling the DKA criteria were included in the analysis, of which 84 (9.0%) patients had concomitant AP. DKA patients with coexisting AP were older (Mean (SD) age of 35.3 ± 14.5 vs. 42.7 ± 12.8 years, p < 0.001) as compared to those without AP. Both groups had a predominance of males (male to female 61.2% vs. 38.8% in the DKA group; 79.8% vs. 20.2% in the DKA and AP group; p < 0.001). Notably, most DKA patients belonged to the Arab ethnicity (54%). However, coexisting AP was most common in the Asian race (65.47%, p < 0.001). More patients in the DKA with AP group had T2D (75%, p < 0.001). No statistically significant difference between mean BMI, duration of DM and co-morbid conditions was observed between the two groups (Table 1).

**Table 1:** Comparison of baseline characteristics between DKA versus DKA with AP patient groups.

Baseline characteristics	Units	DKA alone (852)	DKA + AP (84)	Significance (p-value)
Age, mean ± SD	Years	35.3 ± 14.5	42.7 ± 12.8	< 0.001
Gender	N (%)			
Male		521 (61.2)	67 (79.8)	
Female		331 (38.8)	17 (20.2)	0.001
Ethnicities	N (%)			
Arab		483 (56.7)	23 (27.4)	< 0.001
Asian		253 (29.7)	55 (65.5)	
Africans		90 (10.6)	3 (3.6)	
Others		26 (3)	3 (3.6)	
BMI Median (IQR)	kg/m^2^	23.83 (20.4–28)	25.1 (21.6–27.8)	0.14
Duration of DM, mean ± SD	Years	4.5 ± 6.7	3 ± 5.6	0.06
DM diagnosis	N (%)			
Total T1D		461 (54.6)	21 (25)	< 0.001
Total T2D		383 (45.4)	63 (75)	
Co-morbidities	N (%)			
Dyslipidemia		119 (13.9)	16 (19.2)	0.20
Coronary artery disease		47 (5.5)	7 (8.3)	0.20
Heart failure		10 (1.2)	3 (3.6)	0.10
Chronic liver Disease		29 (3.4)	4 (4.8)	0.34
Hypertension		183 (21.5)	18 (21.4)	0.99
Retinopathy		69 (8.1)	10 (11.9)	0.23
Nephropathy		52 (6.1)	9 (10.7)	0.10

*DKA* Diabetic ketoacidosis, *AP* acute pancreatitis, *BMI* body mass index, *SD* standard deviation, *IQR* interquartile range, *DM* diabetes mellitus, *T1D* type 1 diabetes, *T2D* type 2 diabetes.

### Laboratory investigation results

In comparison to patients with DKA alone, DKA patients with AP had a statistically significant higher mean anion gap (22.9 ± 6.8 vs. 24.4 ± 7.7 mEq/L, p 0.044), median white cell count (WBC) (11.6 (IQR 8.2–17) vs. 15.1 (IQR 10.6–20.6) × 10^3^/µL; p < 0.001) median neutrophil to lymphocyte ratio (NLR) 6 (IQR 2.5–11.2) vs. 7.6 (IQR 4.2–12.7); p 0.01), mean hemoglobin (14.2 ± 2.1 vs. 15 ± 2.4 g/dL; p 0.001), median serum urea level (5.7 (IQR 4–8.1) vs. 7.8 (IQR 4.9–12.4) mmol/L; p < 0.001), median serum creatinine 85 (IQR 64–120) vs. 109 (IQR 78–152) µmol/L; p < 0.001), median total cholesterol 4.3 (IQR 3.3–5.5) vs. 4.7 (3.5–7) mmol/L; p 0.01) and median serum triglyceride (TG) level 1.8 (IQR 1.2–2.5) vs. 2.2 (1.4–5.3) mmol/L; p 0.006) at admission as compared to the patients without AP. DKA patients with AP had lower mean potassium level (3.4 ± 0.5 vs. 3.2 ± 0.6 mmol/L; p 0.001) and lower mean sodium (131.5 ± 4.6 vs. 130 ± 6.6 mmol/L; p 0.048) during the DKA episode. These patients also had a lower mean venous pH at admission (7.15 ± 0.13 vs. 7.09 ± 0.15; p 0.001), median venous pH at 6-h 7.26 (IQR 7.2–7.32) vs. 7.24 (IQR 7.15–7.29); p 0.03) and mean bicarbonate at 6-h (15 ± 5 vs. 13.7 ± 5 mmol/L; p 0.007) in comparison to the DKA only group. There was no statistically significant difference between hbA1c, beta-hydroxybutyrate (BHB) and lactate levels at presentation between the two groups (Table 2).

**Table 2:** Comparison of laboratory parameters between DKA versus DKA with AP patient groups.

Variable	Normal range	DKA alone (852)	DKA + AP (84)	Significance (p-value)
Blood glucose at admission, Median (IQR)	4–11.1 mmol/L	23.3 (18.4–29.8)	25 (17.9–30.4)	0.64
Highest glucose during hospital stay, mean ± SD	4–11.1 mmol/L	12.5 ± 64	142.1 ± 69.1	0.02
HbA1c at admission (mean ± SD)	< 6.5%	12.1 ± 2.8	11.8 ± 2.5	0.41
White cell count at admission, median (IQR)	4–10 × 10^3^/µL	11.6 (8.2–17)	15.1 (10.6–20.6)	< 0.001
Hemoglobin at admission, mean ± SD	12–15 g/dL	14.2 ± 2.1	15 ± 2.4)	0.001
NLR at admission, median (IQR)	NA	6 (2.5–11.2)	7.6 (4.2–12.7)	0.01
Urea at admission, median (IQR)	2.5–7.8 mmol/L	5.7 (4–8.1)	7.8 (4.9–12.4)	< 0.001
Creatinine at admission, median (IQR)	44–80 µmol/L	85 (64–120)	109 (78–152)	< 0.001
Lowest sodium, mean ± SD	135–145 mmol/L	131.5 ± 4.6	130 ± 6.6	0.048
Lowest potassium, mean ± SD	3.5–5.3 mmol/L	3.4 ± 0.5	3.2 ± 0.6	0.001
BHB at admission, median (IQR)	0.03–0.3 mmol/L	5.8 (4.5–7.3)	6 (4.6–7.8)	0.53
Lactate at admission, median (IQR)	0.5–2.2 mmol/L	1.7 (1.1–2.8)	1.9 (1.2–2.7)	0.94
Serum pH at admission, mean ± SD	NA	7.15 ± 0.13	7.09 ± 0.15	0.001
Serum pH at 6-h, median (IQR)	NA	7.26 (7.2–7.32)	7.24 (7.15–7.29)	0.03
Bicarbonate at admission, mean ± SD	22–29 mmol/L	11.1 ± 4.3	9.8 ± 5.7	0.058
Bicarbonate at 6-h, mean ± SD	22–29 mmol/L	15 ± 5	13.7 ± 5	0.007
Anion Gap at admission, mean ± SD	< 10 mEq/L	22.9 ± 6.8	24.4 ± 7.7	0.044
Total cholesterol at admission, median (IQR)	< 5.2 mmol/L	4.3 (3.3–5.5)	4.7 (3.5–7)	0.01
TG at admission, median (IQR)	< 1.7 mmol/L	1.8 (1.2–2.5)	2.2 (1.4–5.3)	0.006
Adjusted calcium, mean ± SD	2.2–2.5 mmol/L	2.39 ± 0.14	2.45 ± 0.15	0.8
Bilirubin, median (IQR)	0–21 µmol/L	11.9 (7–20)	13 (9–22)	0.08
AST, Median (IQR)	0–33 IU/L	23 (16–37)	32 (19–82)	0.003
ALT, Median (IQR)	0–32 IU/L	22 (15–36)	30.5 (19–70)	< 0.001

*DKA* Diabetic ketoacidosis, *AP* acute pancreatitis, *SD* standard deviation, *IQR* interquartile range, *hbA1c* glycated hemoglobin, *NLR* neutrophil to lymphocyte ratio, *BHB* beta-hydroxybutyrate, *LDL* low-density lipoprotein, *TG* triglyceride, *AST* aspartate aminotransferase, *ALT* alanine aminotransferase.

### Clinical outcomes

DKA patients with AP had a longer median length of hospital stay (2.4 (IQR 1.07–4.47) vs. 4.8 (IQR 2.6–7.2) days; p < 0.001), longer median DKA duration (17 (IQR 10–27) vs. 27 (IQR 13.3–41) hours; p < 0.001) and a higher rate of ICU admission (23.1 vs. 39.3%; p 0.001) in comparison to the patients without concomitant AP. In-hospital mortality, 3-month all-cause readmission, 6-month and 12-month DKA recurrence were not statistically significant between the two groups (Table 3).

**Table 3:** Comparison of clinical outcomes between DKA vs DKA with AP patient groups.

Variable	Units	DKA alone	DKA + AP	Significance
Length of stay, median (IQR)	Days	2.4 (1.07–4.47)	4.8 (2.6–7.2)	< 0.001
DKA duration, median (IQR)	Hours	17 (10–27)	27 (13.3–41)	< 0.001
COVID-19 infection	N (%)	10 (1.17)	1 (1.2)	0.1
In-hospital mortality	N (%)	6 (0.7)	2 (2.4)	0.10
Need for admission to ICU	N (%)	197 (23.1)	33 (39.3)	0.001
3-month readmission (all-cause)	N (%)	131 (15.4)	8 (9.5)	0.15
6-month DKA recurrence	N (%)	67 (7.9)	5 (5.9)	0.53
12-month DKA recurrence	N (%)	58 (6.8)	3 (3.6)	0.25

*DKA* Diabetic ketoacidosis, *AP* acute pancreatitis, *IQR* interquartile range, *ICU* intensive care unit.

Univariate logistic regression analysis (Table 4) showed age (OR 1.03 (1.01–1.04), p < 0.001) Asian ethnicity (OR 6.52 (1.95–20.98), p 0.002), male gender (OR 2.5 (1.44–4.33), p 0.001), T2D (OR 3.61(2.16–6.02), p < 0.001), admission WBC count(OR 1.04 (1.02–1.07), p 0.001), hb (OR 1.2 (1.08–1.35), p 0.001), urea (OR 1.04 (1.02–1.07), p < 0.001), creatinine (OR 1.003 (1.001–1.004), p < 0.001), potassium (OR 1.4 (1.09–1.8), p 0.009), venous pH at admission (OR 0.05 (0.01–0.23), p < 0.001), bicarbonate at admission (OR 0.94 (0.89–0.98), p 0.01), anion gap at admission (OR 1.03 (1.0006–1.064), p 0.045), total cholesterol (OR 1.15 (1.06–1.25), p 0.001), LDL (OR 1.19 (1.008–1.40), p 0.03) and TG (OR 1.04 (1.005–1.09), p 0.02) to be the statistically significant factors (p < 0.05) associated with concomitant AP in DKA patients. Multivariate logistic regression analysis (Table 5) revealed only age and total cholesterol level to be associated with the co-existence of DKA and AP.

**Table 4:** Univariate analysis for factors associated with the development of concomitant DKA with AP.

Characteristics (N)	Odds ratio	Significance (p-value)	Confidence interval	
			Upper	Lower
Age	1.032	< 0.001	1.01	1.04
Asian ethnicity	6.52	0.002	1.95	20.98
Male gender	2.50	0.001	1.44	4.33
T2D	3.61	< 0.001	2.16	6.02
White cell count at admission	1.04	0.001	1.02	1.07
Hemoglobin at admission	1.20	0.001	1.08	1.35
Urea at admission	1.04	< 0.001	1.02	1.07
Creatinine at admission	1.003	< 0.001	1.001	1.004
Potassium at admission	1.40	0.009	1.09	1.80
pH vein at admission	0.05	< 0.001	0.01	0.23
Bicarbonate at admission	0.94	0.01	0.89	0.98
Anion gap at admission	1.03	0.045	1.0006	1.064
Total cholesterol	1.15	0.001	1.06	1.25
TG level	1.04	0.02	1.005	1.09
LDL level	1.19	0.03	1.008	1.40

*DKA* Diabetic ketoacidosis, *AP* acute pancreatitis, *LDL* low-density lipoprotein, *TG* triglyceride.

**Table 5:** Odds ratios for factors associated with DKA with AP (multivariate logistic regression analysis).

Characteristics (N)	Odds ratio	Significance (p-value)	Confidence interval	
			Upper	Lower
Age	1.04	0.02	1.003	1.07
Cholesterol	1.2	0.01	1.05	1.4

*DKA* Diabetic ketoacidosis, *AP* acute pancreatitis.

Odds ratios adjusted for gender, admission WBC count, creatinine, venous pH, bicarbonate, anion gap and TG levels.

## Discussion

This retrospective study compared the differences in demographics, biochemical and clinical outcomes of patients with concomitant DKA and AP to those with DKA alone. Patients with DKA and AP were older, predominantly Asians and had T2D. These patients had higher anion gap, WBC count, hb, NLR, urea, creatinine, total cholesterol and TG while a lower venous pH at admission than those with DKA alone (p < 0.05). LOS, DKA duration and ICU admission rate was also higher in patients with DKA and AP than in DKA alone. Age, Asian ethnicity, male gender, T2D, admission WBC count, hb, urea, creatinine, potassium, venous pH, bicarbonate, anion gap, total cholesterol, TGL and LDL level were significant factors associated with the development of coexisting DKA and AP in univariate logistic regression analysis. In multivariate logistic regression analysis, age and total cholesterol level were associated with concomitant DKA and AP.

Studies assessing the prevalence of acute pancreatitis in DKA patients are minimal. A prospective study of 100 DKA patients reported an 11% prevalence of AP in DKA patients^15^. Ma et al. found the prevalence of AP in DKA patients to be 15.53% in a cohort of patients with T2D only^12^. In our cohort consisting of both T1D and T2D patients, 9% of patients with DKA had evidence of concurrent AP. In particular, a statistically significant difference between T1D and T2D groups was observed as 4.1% (21/482) T1D patients, and 13.5% (63/486) T2D patients with DKA had coexisting AP. This highlights the need for particular attention to the presence of this combination in T2D patients.

Early recognition of coexisting DKA and AP is of particular significance as it carries a higher risk of unfavorable outcomes. Madsen et al. described a case of a 27-year-old patient who had a delay in the diagnosis of coexisting DKA with AP and died within 36 h of initial presentation^16^. Ma et al. reported worse biochemical markers in patients with DKA and AP than in DKA alone. Patients with DKA and AP had a lower pH, higher anion gap and evidence of haemoconcentration (high hemoglobin and hematocrit)^12^. Nair et al. also reported a lower pH, higher anion gap and higher blood glucose levels in DKA patients with AP compared to patients without AP^15^. In addition to the similar results concerning the parameters mentioned above, our study also found a higher WBC count, urea and creatinine in patients with DKA and AP. NLR is used widely as a marker of acute stress and increases rapidly following any pathological condition (within 6 h). Normal NLR is between 1–2, and higher values are associated with pathological states, including inflammation and infections^17,18^. King et al. reported an NLR cut-off value of 7.45 or above as predictive of an increased risk of the requirement of intensive care support and death^19^. On the other hand, Liu et al. reported a cut-off of 3.13 or above as predictive of ICU support in patients with COVID-19 infection aged 50 years or above^20^. To our knowledge, NLR in patients with concomitant DKA with AP has not been studied earlier. Our study found a higher NLR (p-value 0.01) in DKA with AP patients compared to the DKA only group indicating increased severity of illness in these patients. A careful assessment of this simple and readily available marker can alert physicians to the possibility of underlying coexisting AP in DKA patients.

An important finding in this study is the greater percentage of T2D (75%) patients in the DKA and AP groups. Noel et al. reported 2.83 fold increased risk of AP in patients with T2D than those without T2D^21^. A meta-analysis conducted by Alexandra et al. also concluded an increased risk of local and systemic complications of AP in patients with DM^22^. This is most likely because patients with T2D have multiple risk factors, including obesity and deranged lipid profile (especially TG levels), that increase the risk of AP. Further studies are needed to understand the relationship between T2D and the risk of AP development.

An interesting aspect of the study was finding a higher percentage of concomitant DKA and AP in patients of Asian ethnicity. Patients of Asian ethnicity comprised 33% of the study population, but 65.5% of patients in DKA with AP group were Asians. The univariate analysis also revealed a statistically significant association between Asian ethnicity and the development of AP with DKA with an odds ratio of 6.52, revealing a higher risk of developing this coexisting condition in this cohort of patients. This could be related to a higher prevalence of metabolic risk factors in the Asian population. Ooi et al. reported lower bicarbonate and higher lactate levels in Asian T2D patients with DKA compared to the White ethnicity^23^. Patients belonging to the Asian race develop metabolic complications at a lower BMI than other racial groups due to a higher percentage of fat at a lower BMI^24,25^. Obesity has also been identified as a risk factor for increased severity and local and systemic complications in AP^26^. An interplay of increased metabolic risk due to high body fat and DM might explain the increased risk of AP in Asians. However, the exact mechanism for developing an increased risk of AP in patients of Asian ethnic group needs to be further assessed in more extensive studies.

### Clinical relevance

Recognition of patients with DKA with AP is clinically relevant due to its impact on patient outcomes and the utilization of healthcare resources. Each DKA admission can cost between $10,000 to $284,000^27^. A longer LOS further considerably increases healthcare costs^28^. Two previous studies comparing DKA patients with and without AP revealed a higher ICU admission rate and a longer LOS in patients with concomitant DKA and AP^12,15^. Our study also confirmed these findings with a longer duration of DKA, a longer LOS and a higher risk of ICU admission in DKA with AP cohort compared to DKA only patients. Knowledge regarding worse clinical outcomes in these patients can help physicians identify and effectively manage these high-risk patients.

### Strengths and limitations

Our study has several strengths. To our knowledge, of the studies comparing DKA patients with coexisting acute pancreatitis to DKA alone, our study has the largest number of patients with concomitant DKA and acute pancreatitis. Furthermore, the inclusion of patients from multiple ethnic backgrounds adds further to the study's strengths. In addition, our study included both T1D and T2D patients with DKA as opposed to a previous study in which patients with T1D were excluded^12^. We strictly followed ADA criteria for DKA diagnosis and Atlanta criteria for AP diagnosis during manual data collection to include patients in the study, thus excluding patients who were wrongly coded as either DKA or AP in the EMR. This robust method of data gathering adds to the authenticity of the study.

Our study has some limitations as well. An important limitation is its retrospective design, due to which adjustment for confounders cannot be performed. Another limitation is the lack of comparison of symptoms between DKA patients with and without pancreatitis. However, DKA and AP have a significant overlap in symptoms, so a comparison of symptoms between DKA versus DKA and AP groups is less meaningful. Severity scores for AP were not calculated and compared as the study involved comparing DKA patients with and without AP. Whether there is any difference in treatment modalities between these two groups of DKA patients was not studied. Larger prospective studies are required to understand the differences between DKA patients with and without AP to provide a higher level of evidence.

## Conclusion

This study showed that patients with concomitant DKA and AP have more severe derangement in the laboratory markers of DKA severity, inflammation, kidney injury and metabolic profile, highlighting much higher severity of illness than patients with DKA alone. Furthermore, this combination also significantly impacts healthcare resources utilization as DKA patients with AP have a longer DKA duration, LOS and requirement for ICU support than DKA patients without AP. More awareness among physicians regarding this combination of life-threatening conditions can lead to early recognition and improved clinical outcomes in patients with DKA.

## Data Availability

The datasets generated and analyzed during the current study are available from the corresponding author on reasonable request.
